# Lest we forget: Dr Paul Farmer (1959–2022) ‘A Global Health Leader at Harvard’

**DOI:** 10.1177/09677720231223501

**Published:** 2024-02-28

**Authors:** Subham Roy, Sakshi Roy

**Affiliations:** 1 Hull York Medical School, 8748 University of York, UK; 2 School of Medicine Dentistry and Biomedical Sciences, 129413 Queen's University Belfast, UK

**Keywords:** Global health, COVID-19, Ebola, public health, human immunodeficiency virus, tuberculosis

## Abstract

In the chronicles of medical advancement, Dr Paul Farmer stands out as a transformative figure whose unwavering commitment to healthcare equity has reshaped treatments for the disenfranchised. An American anthropologist and physician, Farmer has had a profound impact on global health which encapsulates a legacy driven by the steadfast belief that healthcare is an inalienable human right. This article explores Farmer’s monumental contributions, showcasing how his dedication has revolutionized the landscape of healthcare, particularly for those marginalized and underserved. As the architect of Partners In Health and a guiding force at Harvard Medical School, he fostered a novel paradigm of enduring, community-focused medical care. His unyielding advocacy from Haiti to Rwanda confronted entrenched health disparities and galvanized support for increased access to primary and secondary care. His poignant critiques and policy recommendations during the COVID-19 crisis highlighted his relentless pursuit of health justice – advocating for equitable vaccine distribution and tackling racial health disparities. His scholarly works on overlooked health dilemmas and the urgency of global healthcare reflect a legacy that transcends his lifetime. While his passing is deeply felt, Farmer’s visionary ethos continues to inspire, beckoning us toward a more equitable healthcare horizon.

In the chronicles of medical progress, Dr Paul Farmer (Figure 1)^1^ shines as a trailblazing luminary. Dr. Paul Farmer, an American clinician and researcher, co-founded Partners In Health (PIH) and dedicated his career to advancing global health equity. Renowned for his work in providing healthcare to impoverished communities, Farmer's contributions include pioneering community-based healthcare models and advocating for accessible treatment for infectious diseases, particularly in resource-limited settings. His profound impact on healthcare and unwavering commitment to the underprivileged have rendered him a pioneer in the field. Born on 26 October 1959, in North Adams, Massachusetts, and passing away from a sudden cardiac event on 21 February 2022, in Butaro, Rwanda, Farmer’s 62-year life was one of visionary leadership in global health. Lauded for prioritizing the impoverished, his close colleague Joia Mukherjee of Partners In Health (PIH) remembers him as a relentless advocate for equitable healthcare and a moral philosopher who championed uniform standards of care and justice.^1^ His legacy is echoed by Anthony Fauci, the former chief medical advisor to the president of USA, who revered Farmer’s unique zeal and enduring contribution to health equity, celebrating him as an unparalleled icon in public health within this generation.^1^

**Figure 1.**
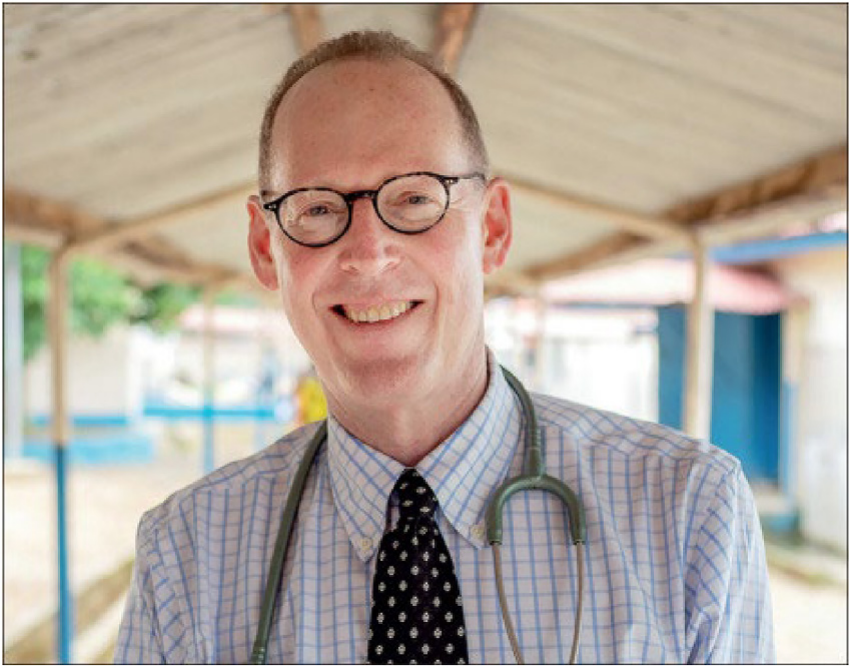
Dr Paul Farmer’s obituary headshot in *The Lancet* (2022), Koidu Government Hospital in Kono District, Sierra Leone.

Paul Farmer’s journey in global health activism began as a volunteer in Haiti following his graduation from Duke University. His early experiences laid the foundation for his life’s work, advocating for the health rights of individuals, including access to medical care. This mission led to the establishment of PIH, which he co-founded in 1987.^2^ Starting with a simple community clinic in Haiti, Farmer’s commitment to sustainable healthcare for the world’s marginalised communities was unyielding. While pursuing his MD and PhD in medical anthropology at Harvard, he remained deeply connected to Haiti, often covertly transporting supplies to support the burgeoning clinic.^3^

Farmer’s philosophy – that health is a fundamental human right – shaped not only his approach to medical practice but also his efforts in education and policy advocacy. His role in PIH evolved into a distinctive model of care: creating lasting health centres within state-run services, staffed mainly by local workers, setting PIH apart from traditional aid organisations. His academic contributions were significant; he led Harvard Medical School’s Department of Global Health and Social Medicine and championed the establishment of institutions like the University of Global Health Equity in Rwanda.

In the global health landscape, Farmer challenged leaders with a critical inquiry: Why does an imbalance in access to information, prevention, and premier healthcare persist across diseases like HIV, tuberculosis, and COVID-19, especially in disparate regions from Haiti to Rwanda. He dissected conventional perceptions of health, delving into the structural and political roots often overlooked in public health discussions. This approach was evident in his final 2 years of writing, where he highlighted our collective failures with candid assessments.

During the COVID-19 pandemic, Farmer foresaw the immense challenge of vaccinating the global populace. At the time of his passing, less than 10% of individuals in low-income countries had received at least one vaccine dose, a stark contrast to the majority in wealthier nations. In response, he advocated for the temporary suspension of intellectual property rights for COVID-19 vaccines, arguing that delays resulted in avoidable deaths.^4^ He also illuminated racial disparities within the United States, in confronting the systemic racial inequities that exacerbate health crises, Paul, alongside his fellow researchers, champions the implementation of racially equitable measures and compensatory initiatives for African Americans.^5^ Such actions are pivotal in mitigating the impact of COVID-19 and other health-related challenges. He articulates the critical imperative to curtail the spread of COVID-19 within US correctional facilities by advocating for widespread vaccination and the strategic reduction of inmate populations, asserting that “our adherence to scientific data and our moral obligation to safeguard those at risk and the wider community compels us to exert our influence to instigate” these necessary reforms.^6^ Furthermore, he underscores the global urgency to ensure the safety of healthcare professionals from the threat of COVID-19 contagion.^7^

Farmer’s activism extended beyond the pandemic. He addressed various neglected health issues, such as reinforcing the global health workforce, acknowledging the long-term effects of Ebola in West Africa, and the health ramifications of poverty in Haiti.^8^ He emphasized the urgent need for palliative care for millions and the moral duty to provide global mental health services, confronting structural violence head-on.^9^ He also acknowledged the ethical imperative to provide global mental health services and identified the systemic injustice frequently inflicted upon individuals with mental illnesses, which arises at the confluence of poverty, marginalization, and bias.

His legacy encompasses more than specific health crises; he offered a holistic view of public health. He criticized the reliance on narrow technological solutions, advocating for the development of comprehensive public health systems attuned to community needs. Under his leadership, the prominent journal, ‘*Health and Human Rights Journal*’ flourished, mirroring his broad interests and deep conviction in the significance of robust health systems. His impact broadened the journal’s audience, reaching professionals and advocates in both the Global North and Global South. He was adamant that the journal should be freely accessible to both readers and authors, ensuring that financial constraints would not hinder the publication or accessibility of quality research.

Although Farmer is no longer with us physically, his influence endures through his scholarly and literary works, inspiring future global health students, practitioners, and policymakers. His passing is a call to action for us to advance his ethos and his belief that a brighter future in healthcare is achievable. His intellectual prowess and compassionate dedication stand as a beacon for future generations to venerate and emulate. Dr. Paul Farmer's body was reportedly contributed to medical science following his death.
